# Is Platelet-rich Plasma Injection more Effective than Steroid Injection in the Treatment of Chronic Plantar Fasciitis in Achieving Long-term Relief?

**DOI:** 10.5704/MOJ.1911.002

**Published:** 2019-11

**Authors:** P Soraganvi, KV Nagakiran, RP Raghavendra-Raju, D Anilkumar, S Wooly, BD Basti, P Janakiraman

**Affiliations:** Department of Orthopaedics, PES Institute of Medical Sciences and Research Kuppam Campus, Kuppam, India; * Department of Community Medicine, PES Institute of Medical Sciences and Research Kuppam Campus, Kuppam, India

**Keywords:** platelet-rich plasma, plantar fascia, steroid injection, plantar fasciitis

## Abstract

**Introduction:** Plantar fasciitis is characterised by pain in the heel, which is aggravated on weight bearing after prolonged rest. Many modalities of treatment are commonly used in the management of plantar fasciitis including steroid injection. Many studies show that steroid injection provides pain relief in the short term but not long lasting. Recent reports show autologous platelet-rich plasma (PRP) injection promotes healing, resulting in better pain relief in the short as well as long term. The present study was undertaken to compare the effects of local injection of platelet-rich plasma and Corticosteroid in the treatment of chronic plantar fasciitis.

**Materials and methods:** Patients with the clinical diagnosis of chronic plantar fasciitis (heel pain of more than six weeks) after failed conservative treatment and plantar fascia thickness more than 4mm were included in the study. Patients with previous surgery for plantar fasciitis, active bilateral plantar fasciitis, vascular insufficiency or neuropathy related to heel pain, hypothyroidism and diabetes mellitus were excluded from the study. In this prospective double-blind study, 60 patients who fulfilled the criteria were divided randomly into two groups. Patients in Group A received PRP injection and those in Group B received steroid injection. Patients were assessed with visual analog scale (VAS) and American Orthopedic Foot and Ankle Society (AOFAS) score. Assessment was done before injection, at six weeks, three months and six months follow-up after injection. Plantar fascia thickness was assessed before the intervention and six months after treatment using sonography.

**Results:** Mean VAS in Group A decreased from 7.14 before injection to 1.41 after injection and in Group B decreased from 7.21 before injection to 1.93 after injection, at final follow-up. Mean AOFAS score in Group A improved from 54 to 90.03 and in Group B from 55.63 to 74.67 at six months’ follow-up. The improvements observed in VAS and AOFAS were statistically significant. At the end of six months’ follow-up, plantar fascia thickness had reduced in both groups (5.78mm to 3.35mm in Group A and 5.6 to 3.75 in Group B) and the difference was statistically significant.

**Conclusion:** Local injection of platelet-rich plasma is an effective treatment option for chronic plantar fasciitis when compared with steroid injection with long lasting beneficial effect.

## Introduction

Plantar fasciitis is a common pathological condition affecting the hindfoot and can often be a challenge for clinicians to treat successfully^1^. It is an overuse injury from repetitive microtrauma that leads to inflammation and local tissue damage^2^. Treatment options include non-surgical management, like non-steroidal anti-inflammatory drug (NSAID) prescription, physiotherapy, night splints and steroid injection, and surgical intervention^1^. The treatment of plantar fasciitis may require a combination of treatment modalities, rather than administering only one treatment at a time^3^. There is no single treatment which has been proven as a gold standard for the treatment of chronic plantar fasciitis. Traditionally, local injection of steroid was used widely for chronic plantar fasciitis treatment. Cochrane review on the use of corticosteroid for plantar fasciitis showed improvement in symptoms at one month, which did not last long^4^. In recent years, platelet-rich plasma (PRP) is being used successfully for the treatment of various chronic tendinitis, including chronic plantar fasciitis. Earlier results of using the PRP to treat plantar fasciitis have been favorable, but there is a paucity of literature where the effectiveness of steroid injection is compared to PRP in chronic plantar fasciitis treatment.

In this study, we compared the efficacy of PRP and steroid injection in the treatment of chronic plantar fasciitis and also analysed the effect of PRP and steroid injection on the thickened plantar fascia.

## Materials and Methods

This study was a randomised double-blind study done at PES Institute of Medical Sciences and Research, Kuppam, Andrapradesh, India, from September 2014 to September 2016. Patients were diagnosed as having plantar fasciitis based on history and clinical examination. The study subjects included all patients with a clinical diagnosis of plantar fasciitis (heel pain lasting more than six weeks) with sonographic evidence (plantar fascia thickness of more than 4mm). Patients with previous surgery for plantar fasciitis, active bilateral plantar fasciitis, presence of vascular insufficiency or neuropathy associated with heel pain, hypothyroidism and diabetes mellitus were excluded from the study. Ethical clearance was obtained from the institution for the study and consent from all patients participating in the study.

The sample size was calculated using a formula based on means and standard deviation as in the study by Jain *et al* using stat software^5^. In our study it was found to be 54 with 27 for each study group. We have rounded it off to 60 with 30 in each group (PRP and steroid groups).

We applied the randomisation method by using sequentially numbered, opaque, sealed envelope technique (SNOSE)^6^. For preparation of envelope, we took 60 identical letter sized envelopes, aluminium foil and single sided carbon paper. Aluminium foil was cut into 60 sheets that are same width as envelope and twice its height, so that when folded it will be same size as envelope. Single sided carbon paper was cut into 60 envelope size sheets. Sixty sheets of standard size papers are taken and each one marked as treatment A (PRP – 30 papers) or treatment B (Steroid – 30 papers) by writing on it. These papers are folded to fit the envelope. One sheet of carbon paper was placed on the top of the folded paper so that carbon side is facing paper. One sheet of aluminium foil was folded over both side of carbon and treatment paper combination. This completed insert was placed in to envelope with carbon paper closest to the front of envelope. The aluminium foil ensures the envelope is opaque and cannot be read by holding it up against strong light source. The carbon paper ensures written matter on envelope is transferred to treatment allocation paper inside envelope. After preparation all enveloped were sealed. These 60 prepared envelopes were shuffled thoroughly and later marked with a serial number over it. All these envelopes were placed in a container in numerical order.

The envelopes were allocated sequentially and participant’s name and other details were entered on the front of the envelope before opening the seal. All patients received treatment as indicated inside the envelope (treatment A – PRP or treatment B - Steroid). A colleague who was not involved in the study did the opening of the sealed envelope and administration of appropriate injection. This method was followed to eliminate bias in the study.

Blood was drawn from patients in both groups for blinding purpose and a screen was used while giving injection so the patients were blinded from the type of treatment they were receiving.

This study included 60 patients with chronic plantar fasciitis. Patients in Group A received PRP (3ml) injection and Group B patients received a steroid injection (Depomedrol 80mg (2ml) + 0.5ml xylocaine 2%). Treatment with NSAIDs was discontinued one week before the injection in both groups. All patients in both groups were advised on plantar fascia stretching exercise.

For PRP preparation, blood was drawn from the cubital vein into six vacutainer tubes, which contained 0.35ml of 3.2% sodium citrate. Vacutainer was centrifuged at 1200 rpm for 10 min in a routine 380 R centrifuge model. Following centrifugation three layers were identified, of which, the bottom layer consisted of red blood cells, the intermediate layer of white blood cells, and upper layer of plasma, platelets, and some white blood cells. The concentrate in the upper layer was carefully collected with a 10cc syringe. The collected volume ranged from 1 to 1.25ml in each vacutainer. Approximately, 1ml of the upper layer of the sample that underwent the first spin step was collected and transferred to one empty 6ml tube. This tube was centrifuged again for 10 min at speed of 2400 rpm (second spin). The upper half of the plasma volume, platelet poor plasma (PPP), was removed. The remaining volume of PRP was used for injection.

Random PRP samples were sent for estimation of platelet count by autoanalyser. Majority of the samples had platelet count of more than 1,000,000/ul in 5ml volume, which was five times the baseline.

Before administration of the PRP or steroid injection, all patients underwent a random blood sugar level assessment. The participants were appropriately counselled before the injection. Injections were given under aseptic condition.

In the PRP group, the injection was given at the site of maximal tenderness using peppering maneuver with 20-gauge-needle after initial instillation of local anesthesia (1ml of 2% plain xylocaine).

In the corticosteroid group, the patients received 2ml (80mg) Depomedrol along with 0.5ml of plain 2% xylocaine using 20G wide bore needle into the point of maximum tenderness. The patients were assessed before injection and during follow-up at six weeks, three months and six months. The assessment was conducted with the visual analog scale (VAS) for pain and the American Orthopedic Foot and Ankle Society (AOFAS) score for function. Physiotherapy colleagues who did not have access to the treatment/injection data did scoring. The thickness of plantar fascia was assessed at the 6th-month follow-up visit using ultrasound.

Statistical analysis, was done using SPSS 11 software. Independent t-test was used to compare the mean difference between the two groups, paired t-test was used to compare the mean difference between before and after paired data. The correlation was done for continuous variables comparing the efficacy of intralesional corticosteroid with autologous platelet-rich plasma injections in the management of chronic plantar fasciitis.

## Results

Out of the 60 patients one patient from group A (PRP group) and two patients from group B (steroid group) were lost to follow-up, and the results in the remaining 57 patients were analysed. There were 31 female and 26 male patients in the final study group. Mean age of the patients in Group A and Group B was 40.27 and 39.35 years, respectively. The right heel was affected in 29 patients and left in 28 patients (Table I).

**Table I:** Comparison of the characteristics of both groups

	PRP Group (29)	STEROID Group (28)
Male	14 (48%)	12 (57%)
Female	15 (52%)	16 (43%)
Age	40.27yrs (mean, SD – 8.03)	39.35yrs (mean, SD-12.5)
Right Side	14 (48%)	15 (54%)
Left Side	15 (52%)	13 (46%)

**Fig. 1:**
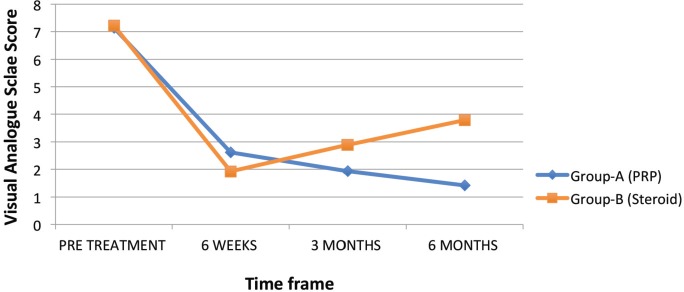
Representation of VAS scores before treatment and at interval of six weeks, three months and six months after treatment.

Mean visual analog scale score in Group A and Group B before injection was 7.14 and 7.21 respectively, which improved to 2.62, 1.93 and 1.41, respectively, in Group A at six weeks, three months and six months of follow-up. In Group B mean VAS improved to 1.93 at six weeks follow-up, and 2.89 at three months and 3.76 at six months follow-up (Fig. 1). The difference between the two groups was statistically significant at six weeks (p<0.007), three month (p<0.001) and six months (p < 0.001) (Table II).

**Table II:** Mean VAS score in both groups

VAS	Group A (PRP)	Group B (Steroid)	P value (at end of 6 months follow-up)
Pre-Treatment	7.137	7.214	
6 Weeks	2.62	1.928	
3 Months	1.931	2.89	
6 Months	1.413	3.785	<0.001

**Fig. 2:**
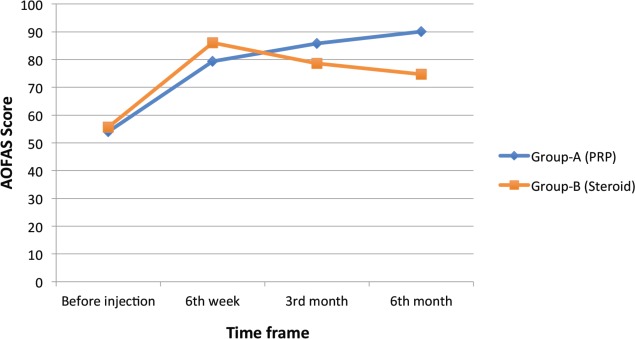
AOFAS before treatment and at different follow-up visits in both groups.

Mean AOFAS score in Group A and Group B before injection was 54 and 55.63 respectively. AOFAS score improved to 79.3, 85.72 and 90.03 in Group A and 86.06, 78.57 and 74.67 in Group B, respectively, at six weeks, three month and six months follow-up (Table III). In Group B, the score showed significant increase initially which decreased at the third-month and six-month follow-up, whereas in Group A there was an increase in the score at follow-up visits (Fig 2). The difference between the two groups was statistically significant at the six-month follow-up (p< 0.001) (Table III).

**Table III:** AOFAS (mean) in both groups

PARAMETER	Group A (PRP)		Group B (Steroid)		P value (at end of 6 months follow-up)
	Mean	S.D	Mean	S.D	
AOFAS Before injection	54	4.117	55.63	4.344	
6th Week	79.3	2.355	86.06	2.686	
3rd Month	85.72	2.361	78.57	1.913	
6th Month	90.03	3.366	74.67	3.693	<0.001

Prior to injection the average thickness of plantar fascia assessed using ultrasound was comparable in both the groups (5.78mm in Group A and 5.6mm in Group B, respectively). At the post-treatment sonographic evaluation at six months following the injection, Group A had significant reduction (mean 3.35mm, 35.45%) in the thickness of plantar fascia as compared to Group B (mean 3.75mm, 29.16%), (Table IV). The difference between the two groups was statistically significant at six months (p < 0.0003).

**Table IV:** Thickness of plantar fascia before treatment and at 6 months after treatment

VARIABLES	Average plantar fascia thicknesses in each group				P value
	Group A (PRP)		Group B (Steroid)		
	Mean	S.D	Mean	S.D	
Before injection	5.78	0.592	5.60	0.556	<0.5562
6th month Post-injection	3.35	0.409	3.75	0.404	< 0.0003

## Discussion

Plantar fasciitis is a degenerative soft tissue condition that occurs near the site of origin of the plantar fascia at the medial tuberosity of the calcaneus^7^. In chronic cases normal fascia is replaced by angiofibroblastic tissue^7^. Historically plantar fasciitis was assumed to be an inflammatory process. Histological findings like chondroid metaplasia, calcification, and collagen necrosis suggest a degenerative mechanism^7^. Hence, the term fasciosis was used by many authors rather than fasciitis. Plantar fasciitis is usually a self-limiting condition and non-operative method is usually successful. However, few patients develop chronic plantar fasciitis where pain persists and certainly affects the day-today quality of life of the patients.

Many treatment modalities have been in practice, among which corticosteroid injections have been extensively used, but only seemed to be useful in the short term and only to a small degree^8^. Potential complications associated with steroid injection raise concern about benefit against the risk involved in steroid injection. Histological studies have indicated plantar fasciitis as a degenerative disorder, hence prostaglandin mediated anti-inflammatory action of steroid is unclear. However, inhibition of fibroblast proliferation and expression of ground substance proteins by corticosteroids may be the possible explanation for the beneficial effect of steroid injection^9^.

Various studies have shown that platelet-rich plasma injection as an effective treatment option for chronic plantar fasciitis^10-19^. Plantar fasciitis is considered a degenerative tissue condition due to micro-tear in fascia rather than inflammation. This results in denaturation of collagen and angiofibroblastic hyperplastic tissue is seen in histology^7^. PRP is rich in growth factors like transforming growth factor, vascular endothelial growth factor, and platelet-derived growth factor and inflammatory mediators like cytokines and interleukins, such as interleukin 4, 8, 13, interferon-α, and tumor necrosis factor-α^7^. The concentration of these factors is low in the plantar fascia due to hypovascularity and hypocellularity^7^. PRP delivers growth factors along with platelets directly to the site of the lesion, since all these factors affect healing stages necessary to reverse chronic plantar fasciitis^7^. Alpha particles of platelets release stored platelet-derived growth factors after stimulation. It increases fibroblast migration and proliferation and improves collagen deposition, which promotes angiogenesis and fiber repair^7^.

Literature on treatment options show a variable outcome when PRP and steroid injection are used in the treatment of chronic plantar fasciitis. Some studies found PRP to be more effective whereas others did not find a significant difference in the outcome^10, 12, 13^. When steroid injection was compared with autologous blood injection in a study by Lee *et al,* they found that the corticosteroid group had significantly lower VAS than autologous blood group^14^. Monto *et al* comparing PRP and corticosteroid injection in the treatment of failed non-surgical treatment of plantar fasciitis, concluded that a single injection of PRP improved pain and function more than steroid injection and beneficial effects sustained for a longer time^10^.

In our study, we compared the effectiveness of PRP and steroid injection in patients with chronic plantar fasciitis where other conservative treatments had failed. We adopted PRP preparation as per Amanda *et al* recommendations^20^. Two spin method showed higher growth factor levels and higher platelet counts. In our study, most of the samples had platelet counts of more than 1,000,000/ul in 5ml volume, which is five times the baseline. Peppering technique of injection was found to be more effective and was used in our study^16-18^. In this technique, fascia is injected at multiple sites through a single skin portal^16-18^. The injection was administered at the point of maximum tender points. Benefits of injection under ultrasound guidance are doubtful. Ultrasound-guided administration of injection was used in some studies^21, 22^. However, studies have reported no significant difference between ultrasound-guided injection and injection at the tender spot^23^.

There are various methods of preparation of platelet-rich plasma. In each method of preparation, platelet concentration varies. In present literature, there is paucity of information regarding the more superior type of method of preparation. Also, the effectiveness of leukocyte-reduced or rich PRP preparation is debatable^24, 25^. In our study, leucocytes were not filtered from PRP. Activation of PRP initiated during the preparation process by degranulation of platelets^26^. Adding thrombin or calcium chloride activates PRP. Spontaneous platelet activation occurs after exposure to native collagen^27^. Presently, available literature lack evidence of the most suitable method of activation of PRP. The beneficial effect of activating PRP before the injection is not supported by all studies. All patients in our study received freshly prepared PRP. We have not used any agent to activate PRP.

Jain *et al* in their study comparing single injection of PRP and steroid injection in chronic plantar fasciitis, found no significant difference in functional outcome in both groups at six months follow-up^28^. Similar results were also observed in other studies^29^, whereas many studies have shown the long-lasting beneficial effects of PRP when compared to steroid injection with improved AOFAS score and VAS score^5, 10, 30^.

In our study, we observed that in both PRP and steroid injection group, VAS and AOFAS score improved after one injection and improvement in pain and AOFAS score was more in the steroid group compared to PRP group at first follow-up visit. On later follow-up both VAS and AOFAS score in PRP group continued to improve and at the end of six months follow-up the PRP group showed better improvement compared to steroid group and improvement in score was statistically significant. The decline in pain and function scores of steroid group after six weeks suggest that steroid injection is more effective only for short-term relief. The mechanism of reduction in pain and improvement in the function after PRP injection is not clear. PRP contains hepatocyte growth factor (HGF) along with other growth factors. The anti-inflammatory action of HGF is mediated by disrupting the nuclear factor kappa B (NF-kB) transactivating activity, which results in decreased expression of COX-1 and COX-2 genes. By this action, HGF is known to protect tissues from inflammatory damages. Thus, the anti-inflammatory action of PRP is through HGF. This explains the initial improvement in VAS score and reduction in pain following PRP injection^31^.

Studies have shown a significant reduction in plantar fascia thickness after PRP injection^18, 20^. In our study, plantar fascia thickness in both the PRP group and corticosteroid group were comparable prior to injection. However, at six months follow-up, the PRP group had a significant reduction (35.45%) in the thickness of plantar fascia compared to corticosteroid group (29.16%). The difference between the two groups was statistically significant.

Limitation of this study is the variability of platelet concentration among different patients. Lack of standardisation in preparation, concentration of platelets and dosage were barriers for critical evaluation. Further basic research is necessary in this field for understanding the exact mechanism of action of PRP. However, the results of PRP injection as a biological modality of treatment in orthopedic conditions are encouraging.

## Conclusion

Local injection of platelet-rich plasma is an effective treatment option for chronic plantar fasciitis when compared to steroid injection and beneficial effects are long-lasting.
